# Automated Classification of Whole Body Plethysmography Waveforms to Quantify Breathing Patterns

**DOI:** 10.3389/fphys.2021.690265

**Published:** 2021-08-20

**Authors:** Michael D. Sunshine, David D. Fuller

**Affiliations:** ^1^Rehabilitation Science Ph.D. Program, University of Florida, Gainesville, FL, United States; ^2^Department of Physical Therapy, University of Florida, Gainesville, FL, United States; ^3^Breathing Research and Therapeutics Center, University of Florida, Gainesville, FL, United States; ^4^McKnight Brain Institute, University of Florida, Gainesville, FL, United States

**Keywords:** whole body plethysmography, cluster, waveform, opioid, ampakine

## Abstract

Whole body plethysmography (WBP) monitors respiratory rate and depth but conventional analysis fails to capture the diversity of waveforms. Our first purpose was to develop a waveform cluster analysis method for quantifying dynamic changes in respiratory waveforms. WBP data, from adult Sprague-Dawley rats, were sorted into time domains and principle component analysis was used for hierarchical clustering. The clustering method effectively sorted waveforms into categories including sniffing, tidal breaths of varying duration, and augmented breaths (sighs). We next used this clustering method to quantify breathing after opioid (fentanyl) overdose and treatment with ampakine CX1942, an allosteric modulator of AMPA receptors. Fentanyl caused the expected decrease in breathing, but our cluster analysis revealed changes in the temporal appearance of inspiratory efforts. Ampakine CX1942 treatment shifted respiratory waveforms toward baseline values. We conclude that this method allows for rapid assessment of breathing patterns across extended data recordings. Expanding analyses to include larger portions of recorded WBP data may provide insight on how breathing is affected by disease or therapy.

## Introduction

Whole body plethysmography (WBP) chambers enable collection of waveform data corresponding to breathing as well as related behaviors such as sighing or sniffing. The WBP method is an important tool in biomedical research, and is used extensively in preclinical studies of breathing on unrestrained and non-anesthetized animals (van den Hoogen and Colpaert, 1986; Bavis et al., 2011; Nicaise et al., 2013; Hill et al., 2020). In most WBP recording conditions, mammals do not breathe with the metronomic patterns that typify breathing under anesthesia or *in vitro*. Rather, the respiratory-related waveforms recorded using WBP in awake animals are dynamically changing. Further additional factors such as posture (Freedman, 1979), airway resistance (Lofgren et al., 2006), thoracic cavity stiffness, vagal feedback (Sammon and Bruce, 1991; Del Negro et al., 2002), temperature, humidity, and body movement can affect respiratory airflow measured with a WBP. Due to variability in WBP waveforms, large portions of the recording periods are usually omitted from WBP waveform analysis in favor of arbitrarily defined periods of baseline or “stable breathing.” Analysis of these periods are usually limited to assessment of inspiratory tidal volume (V_T_) and respiratory rate.

The first purpose of the current study was to develop a principle component based algorithm for rapid quantification of WBP waveforms across extended periods of data recording. Our rationale was that the assessment of respiratory rate and V_T_ from specific segments of a data record does not extract all relevant information from a prolonged recording of breathing. Variations in the temporal appearance of respiratory waveforms not detectable with standard analyses of rate and V_T_ could prove valuable when assessing respiratory control, particularly in disease models. Several studies have suggested that reduction in the variance of respiratory output (i.e., fewer degrees of freedom in the respiratory system) can be a predictor of underlying pathology and poor patient outcomes (Wysocki et al., 2006; Papaioannou et al., 2010). The ability to produce a wide range of respiratory-related behaviors is a signature of a healthy respiratory neuromuscular system (Bruce, 1996; Papaioannou et al., 2011). Another important consideration is that detailed analysis of WBP recordings in their entirety (e.g., minutes to hours) will remove potential bias that results from arbitrary selection of small segments of recorded data. To enable rapid evaluation of the temporal aspect of WBP-derived respiratory waveforms we created an analysis algorithm using MATLAB. Waveforms were first separated into four time domains then clustered based on inner squared Euclidean distance, the number of clusters was determined by adapting Youden’s index (Youden, 1950), to determine the point where less information is explained with the addition of more clusters (Thorndike, 1953). Thus, each waveform was assigned to a cluster, which allowed us to track when and how often breaths in these clusters occurred.

The second purpose of our study was to use the new WBP waveform analyses algorithm to evaluate breathing in adult rats after opioid overdose and treatment with a rescue drug known to stimulate breathing. Hypoventilation following opioid overdose is a major medical problem and results in a high number fatalities each year in the United States (Seth et al., 2018) and around the world (Csete et al., 2016; Bedene et al., 2020; Bird and Robertson, 2020; van Amsterdam et al., 2020). Accordingly, preclinical studies of the mechanisms of opioid-induced respiratory depression are receiving increased attention, and WBP is a frequently used tool in this research (Yassen et al., 2008; Chevillard et al., 2009; Ren et al., 2009; Baby et al., 2018). Accordingly, methods which provide high throughput analyses of WBP waveforms after opioid overdose could be of widespread utility.

To reverse opioid-induced respiratory depression, we used an ampakine (Lynch, 2006; Arai and Kessler, 2007) that can augment α-amino-3-hydroxy-5-methyl-4-isoxazolepropionic acid (AMPA) receptor mediated neurotransmission. Glutamate-mediated neurotransmission, acting via AMPA receptors, is a primary driver of brainstem respiratory rhythm generation and makes a substantial contribution to excitation of spinal respiratory motor neurons (Chitravanshi and Sapru, 1996; Rana et al., 2019a,b). Ampakines are class of drugs that alter AMPA receptor channel kinetics (Lynch, 2006; Arai and Kessler, 2007), and can generally be classified as type I (“high impact”) and type II [“low impact”; reviewed in Arai and Kessler (2007)]. The drugs first described in the laboratories of Lynch and Rogers (Palmer et al., 1997) are considered high impact based on the observed effect on AMPA channel kinetics. Low impact ampakines retain the ability to act as positive allosteric AMPA receptor modulators, but the receptor kinetics have a shorter decay time constant (Arai and Kessler, 2007). Prior work shows that a low impact ampakine with relatively low water solubility, CX717, can effectively stimulate breathing after opioid-induced respiratory depression induced in rats (Ren et al., 2009) and humans (Oertel et al., 2010). A recent study tested a water soluble ampakine, CX1942, and found that it could stimulate breathing in goats following an opioid overdose (Haw et al., 2016). Here we tested the hypothesis that following opioid overdose in rats, CX1942 could restore temporal breathing patterns, in a dose-dependent fashion, toward baseline values. The newly developed analyses algorithm afforded the opportunity to explore the impact of CX1942 on WBP waveforms beyond the evaluation of rate and amplitude that used in prior studies (Lu et al., 2006; Ren et al., 2009; Joshi et al., 2019).

## Materials and Methods

### Experimental Data Collection

Experiments conformed to the ARRIVE guidelines and the regulations set forth by the Institutional Animal Care and Use Committee at the University of Florida, which approved all procedures. Animals were housed on a 12/12 light dark cycle, lights on at 7 am. Male Sprague Dawley rats (*n* = 32; 371 ± 20 g) were briefly sedated by exposure to 2.5% isoflurane in O_2_, and up to two tail vein catheters were placed as dictated by the experimental protocol. The catheters consisted of PE-10 tubing connected to a Terumo Surflo injection plug. Catheters were preloaded so that the dead space was filled with the necessary drug (e.g., ampakine CX1942, fentanyl). Following tail catheter placement, rats were returned to their cages and placed in an E-collar and continuously monitored to ensure they did not chew or scratch the catheter tubing. After 1 h, rectal temperature (38.4 ± 0.6°C) was measured and rats were placed in the plethysmography chambers (Buxco PLY 4213 chamber, with TRD5700 transducer amplified with a Max2275 strain gauge amplifier). The plethysmography recordings were conducted between 9 am and 3 pm. Sleep wake state was not assessed during this study. The tail vein catheters were funneled through a hole in the plethysmograph chamber that was then sealed. Thus, drugs could be infused continuously while breathing was monitored. Plethysmography data were recorded under normoxic conditions (21% O_2_, balance N_2_) using a Buxco FinePointe system sampled at 500 samples per second. The rats were not restrained during these procedures and were free to move within the chamber. Following an extended period of baseline breathing, fentanyl was administered intravenously until tidal volume (V_T_) was reduced to 50% of baseline values. Once tidal volume was reduced to 50% of baseline values, either ampakine CX1942 or saline vehicle (sham) were injected intravenously. Each experimental group consisted of eight rats that received an infusion of either saline or ampakine CX1942 at one of three doses: 3, 10, or 30 mg/kg. Fentanyl is a potent respiratory suppressant and the dose was titrated to suppress tidal volume to 50% of baseline values. The fentanyl dose required to decrease V_T_ by 50% ranged from 60 to 240 mcg/kg (109 ± 51 mcg/kg). Fentanyl dosing began at 60 mcg/kg, with the exception of two rats in the intravenous 10 mg/kg ampakine CX1942 group that started with 30 mcg/kg fentanyl. In those two cases, 30 mcg/kg was insufficient to suppress ventilation and the dose was increased to 60 mcg/kg after 12 min. The fentanyl dose was increased to 120 mcg/kg (cumulative dose) in 20 rats (*n* = 2 in saline, *n* = 8 in 3 mg/kg, *n* = 3 in the 10 mg/kg, *n* = 7 in the 30 mg/kg group). Further, three rats (one in each group except the 10 mg/kg ampakine CX1942 group) required a 240 mcg/kg cumulative dose to achieve a 50% reduction in ventilation. Rectal temperature measured at the end of the experiment was 38.0 ± 0.9°C which represented an average drop of 0.4°C. The small reduction in body temperature observed here was anticipated as opioids are known to cause a sedative effect and reduce body temperature (Geller et al., 1983). Ampakine CX1942 treatment did not impact body temperature and thus the drop was similar across all groups as evaluated using two-way repeated measures analyses of variance (ANOVA): treatment (saline, 3, 10, and 30 mg/kg CX1942; *p* = 0.244); time (pre/post; *p* = 0.009); treatment × time interaction (*p* = 0.573). The equations used for calculating tidal volume in whole body plethysmography incorporate body temperature to correct for these changes (Drorbaugh and Fenn, 1955).

### Data Analysis and Waveform Processing

**FIGURE 1:**
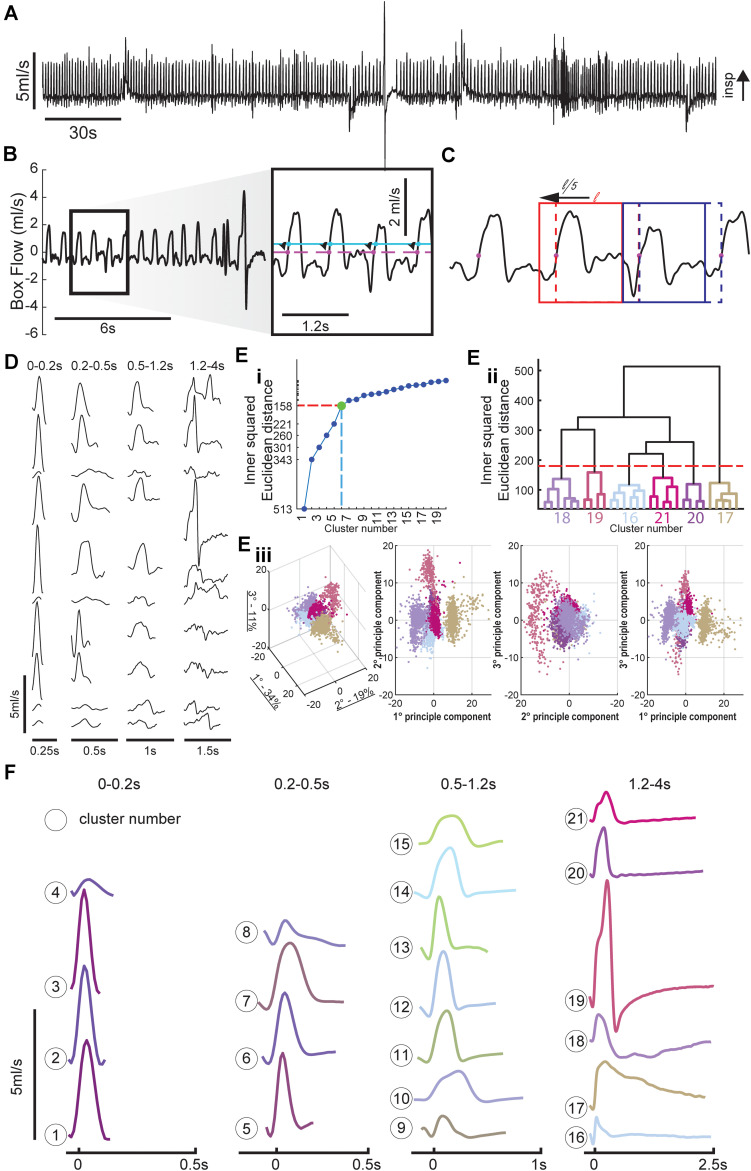
Method for breath detection and respiratory waveform clustering. **(A)** Example plethysmography flow trace. **(B)** Inspiratory efforts were detected with a threshold of 0.1 ml/s to avoid accidental noise detections, the starting point of each breath was then set to the previous zero-crossing and ended at the start of the next breath. **(C)** Breath detection windows were shifted back in time 20% of the breath length (max of 100 ms) to capture the entire inspiratory effort. Two windows are shown at their original position (dashed box) and final position (solid line). Positive values indicate inspiratory activity. Breaths were separated into four time domains to allow for cluster sorting; these time domains correspond to distinct respiratory behaviors. **(D)** Example waveforms from several animals for each of the four time domains (sniffing, short tidal, long tidal, and very long breaths). **(E)** Cluster methodology. Long duration (1.2–4 s) breaths are used as an example to illustrate clustering method. The relationship between cluster number and variance is plotted to show how we determined the number of clusters within each time domain. A 30 cluster hierarchical linkage tree with the six cluster cut-off is shown to illustrate the variance between the clusters (height of line connecting the six clusters), and variance within clusters (height of lines in color coded subtree). Every breath with the prolonged breath duration is plotted in principle component (PC) space the amount of variance explained by the eigenvector is noted in the 3D PC space in the axis labels, the colors of the breath scatter points matches their assigned cluster. **(F)** Average waveforms for the 21 clusters, the average waveform length is equal to the mean + 1 standard deviation (SD) of the lengths of all waveforms within that cluster.

MATLAB code was written to detect distinct waveforms as discussed throughout the “Results” section. Detailed signal processing information is included here. The MATLAB code is freely available upon request. The flow waveforms (Figure 1A) were processed with a 2nd order butterworth 15 Hz lowpass filter and then downsampled to 90 samples per second. Within the continuous data record, individual waveforms were first identified via a threshold crossing method. Specifically, a threshold value of 0.3 ml/s (Figure 1B, blue line) was used to avoid detecting noise fluctuations around zero, evidenced in the inset of Figure 1B. Inspiratory epochs were then shifted to the preceding zero-crossing (Figure 1B, pink dots) to mark the true beginning of inspiration. To be certain that the onset of the inspiratory effort was captured, the breath detection window was shifted back 20% of the calculated breath length (max of 100 ms, Figure 1C). A matrix consisting of all waveforms, from all animals was then compiled (Supplementary Figure 1A). Waveforms were then grouped into four time domains (0–0.2, 0.2–0.5, 0.5–1.2, and 1.2–4 s, Figure 1D and Supplementary Figure 1B). Assignment to a particular time domain was based duration of the waveform, defined as the period from the onset of inspiration to the subsequent inspiration. The four time domains encompassed respiratory-related behaviors including sniffing, tidal breathing, and augmented breaths. The characteristics of augmented breaths (also referred to as sighs) has previously been discussed in detail (Cherniack et al., 1981; Golder et al., 2005). Short duration waveforms were classified as sniffing when the total cycle duration was below 0.2 s in duration. This is in line with previous work showing that sniffing to sample odors occurs at cycle durations between 100 and 250 ms (Kepecs et al., 2007; Wesson et al., 2009). The length of each waveform varied slightly within each time domains, and to enable the subsequent principle component analysis (PCA) it was necessary to “pad” zeros to the end of each waveform to ensure that the same number of samples were used. Within each time domain a matrix consisting of the flow rate at each sample for all waveforms was compiled and then analyzed using the PCA function in MATLAB (Supplementary Figure 1C). PCA is a well-established mathematical procedure that reduces data set dimensionality; details of the underlying mathematics are well established (Krzanowski, 2000; Jolliffe, 2002; Jackson, 2003; Seber, 2004). In brief, PCA analysis produces an Eigenvector for each column within the dataset and a value for how much variance is explained by each Eigenvector. We calculated how many Eigenvectors were required to explain more than 90% of the variance in the data. This was done by cumulatively summing the variance explained by each Eigenvector starting with the first and continuing until the variance explained was greater than 90%. A hierarchical cluster tree was then constructed from these Eigenvectors. This MATLAB linkage function utilizes a “bottom-up” approach in which each waveform is initially assigned to its own independent cluster. In turn, that cluster is then associated with the next waveform that is determined to be most similar by determining the shortest pairwise distance between each waveform. The method allows the cluster tree (Figure 1E) to be segmented into nodes with values that range from a low of 1 and up to the number of waveforms in the sample.

There was no *a priori* way of determining the number of nodes within each time domain, we set the number of clusters within each of the four time domains using the principle of Youden’s index for receiver operating characteristic (ROC) analysis (Thorndike, 1953). If the distance between clusters is plotted relative to number of clusters in the space, an “elbow” is often apparent in the data plot. This occurs because the average cluster distances decrease as more clusters are added to the space, and is taken to indicate that correct number of clusters in the space has been identified (Thorndike, 1953; Noto et al., 2018). In our dataset we identified 4, 4, 7, and 6 clusters, respectively, to the 0–2, 0.2–0.5, 0.5–1.2, and 1.2–4 s time domains. To establish the final cluster assignments of each waveform, we used the MATLAB dendrogram function (Espinoza et al., 2012). The dendrogram function illustrates the relationship between each set of clusters as shown in Figure 1E, the height in the cluster tree represents the distance between each cluster. This procedure also creates a plot to show the similarity of waveforms both within and between clusters. The dendrogram function also assigns a node (cluster) number to each row of the corresponding input; this was used as the final cluster number. Each waveform in the original matrix, consisting of all identified waveforms from all animals, was then assigned a cluster number (Supplementary Figure 1D). This allowed us to annotate flow traces, generate raster plots, and calculate the prevalence of each cluster number across experimental timeline and between groups.

Respiratory rate and V_T_ was calculated over the final 5 min of each period. The respiratory rate was determined by counting the number of breaths detected within the 5 min period and dividing by the duration in minutes, note; in one animal the rescue period was less than 5 min. The breath snippets, which consist of flow waveforms were integrated to covert the flow into volume. An average V_T_ was then calculated for the 5 min period. Minute ventilation was calculated as the product of the respiratory rate and tidal volumes.

All statistical tests were performed in MATLAB 2019a, and the specific test is noted in the results and/or figure legends. Data from all 32 animals was pooled for in comparisons that assessed the impact of fentanyl compared to baseline as the rescue drug had not been administered, in figures showing the impact of the rescue drug data is stratified by treatment group. Data in text and figures are presented as mean and standard deviation.

## Results

### Principle Component Analysis Allows for Rapid Classification of Respiratory Waveforms

Figure 1A shows an example of airflow traces recorded using WBP. Note the highly varied waveforms, which include tidal breathing, sniffing, and an augmented breath (i.e., a sigh) (Li et al., 2016). Example waveforms from several animals and multiple time points illustrate the variability within each time domain (Figure 1D). These time domains encompassed sniffing (0–0.2 s), short (0.2–0.5 s), and long (0.5–1.2 s) tidal breaths and extended waveforms (1.2–4 s) which typically included a prolonged expiratory duration (T_E_). The total number of waveforms sampled across the four time domains was 127,602, 67,584, 125,232, and 12,727, respectively.

The hierarchical linkage tree and PCA space within the 1.2–4 s time domain shows six distinct clusters (Figures 1E_**ii**_,E_**iii**_). Similarly, there were determined to be four clusters in the 0–0.2 s time domain, four clusters in the 0.2–0.5 s time domain, seven clusters in the 0.5–1.2 s time domain. The average of all the waveforms within each cluster are plotted in Figure 1F. Importantly, there were no waveforms within a cluster that were observed only in an individual animal, rather clusters generalized across all animals. This confirms that the clustering paradigm was not simply attributing breaths from an animal to its own cluster.

Each of the waveform shapes shown in Figure 1F were empirically determined to be different based on the aforementioned analysis, but some clusters likely represent subtle variants of the same overt behavior. For example, clusters 1–3 in the shortest time domain represent slight variations on presumed sniffing-related waveform; however, cluster 4 is distinct and may represent artifactual threshold crossings. Clusters in the middle two time domains (0.2–0.5 and 0.5–1.2 s) consisted of variations on the typical inspiratory tidal breath. In particular certain clusters (e.g., 12 and 13) have a brief period of negative flow prior to inspiration which is absent in other clusters (e.g., 10 and 15). Figure 1C shows the impact of implementing the sliding window for purposes of better capturing the entire respiratory waveform. The dashed line in Figure 1C indicates the initially detected threshold crossing. The second breath in the example (highlighted by the blue box) illustrates how a period of negative flow (downward deflection) was captured when the evaluation window was slid back. Within the clusters of the longest duration waveforms (i.e., 1.2–4 s), the classic augmented breath was evident from the two-phase inspiration followed by apnea seen in cluster 19 (Figure 1F). This time domain also contained what appear to be eupneic tidal breaths followed by spontaneous apneas (e.g., clusters 20 and 21).

### Fentanyl Infusion Alters Respiratory Behaviors and Cluster Prevalence

**FIGURE 2:**
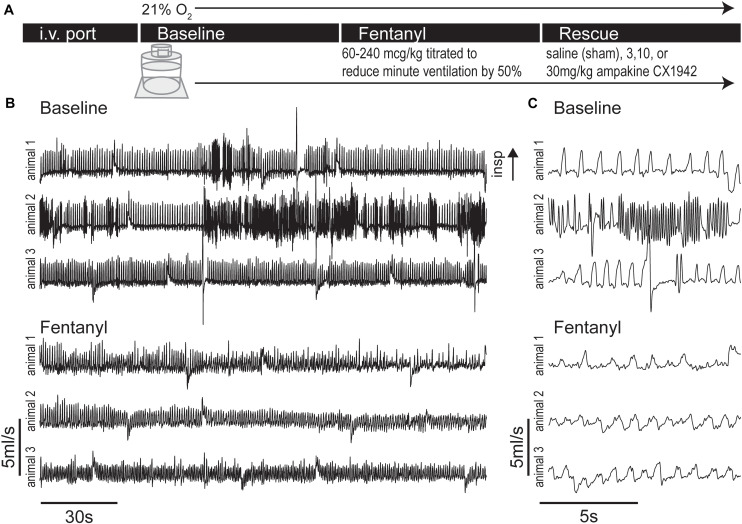
Experimental protocol and example plethysmography traces during baseline and fentanyl periods. **(A)** Experimental timeline, rats were lightly anesthetized with isoflurane to place an i.v. port in the tail vein, then rats were placed in whole body flow through plethysmography chamber (21% O_2_ balance N_2_), after a 32 ± 7 min (mean ± SD) baseline period fentanyl was infused until minute ventilation dropped to 50% of baseline levels. A rescue dose (3, 10, or 30 mg/kg) of ampakine CX1942 or control (saline) was then injected. **(B)** Example flow traces showing the baseline and fentanyl period from three rats. Under baseline conditions, the varied nature of rat respiration produced waveforms indicative of switching between multiple behaviors (e.g., quiet breathing and sniffing). However, injection of fentanyl caused respiratory waveforms to exhibit less variation and decrease in amplitude. **(C)** Expanded traces from panel **(B)**.

To assess the impact of opioids on the temporal appearance of WBP waveforms we recorded breathing during baseline conditions and following fentanyl infusion via the tail vein in unanesthetized rats (Figure 2). Overall, 333,145 breaths were processed across 32 rats from >50 h of WBP recordings, which encompassed the entire experimental timeline.

**FIGURE 3:**
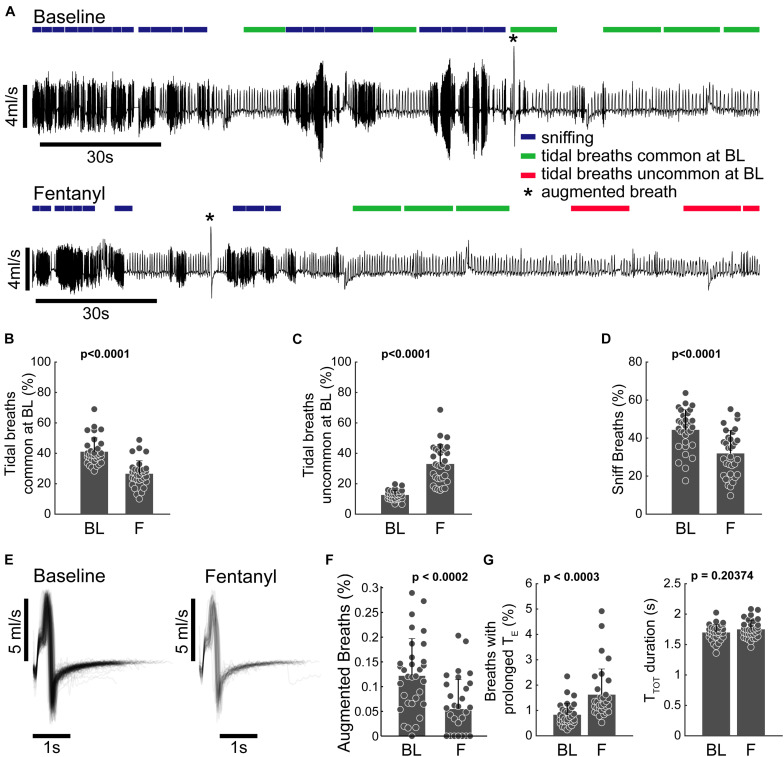
Clustering allows for behavioral annotation and quantification. **(A)** Example plethysmography traces from the baseline and fentanyl periods of the same animal. 20-breath bins are annotated to indicate sniffing (blue), common tidal breathing (green), uncommon tidal breathing (red), and augmented breaths (*cluster 19). Epochs were only marked if the majority of breaths met the inclusion criteria (the majority of waveforms belonging to a specific set of clusters, see text). **(B,C)** Percentage of breaths belonging to clusters 5–15 that represent breaths that have lengths associated with tidal breathing. **(B)** Breaths that had waveforms common at BL (clusters 5, 6, 11, 12, and 14) decreased following fentanyl infusion and **(C)** breaths with waveforms uncommon at baseline increased during fentanyl infusion. **(D)** Percentage of breaths belonging to clusters 1–3 (sniffing behavior) during the baseline and fentanyl periods. **(E)** Pile plot of all breaths classified as belonging to cluster 19 during baseline and fentanyl infusion. **(F)** Percentage of breaths during baseline (BL) and fentanyl infusion (F) which were classified as cluster 19. Cluster 19 breaths were completely absent during the last 3rd of the fentanyl infusion period (i.e., by the time the rat’s minute ventilation dropped to 50% of baseline there were no augmented breaths). **(G)** Prevalence, and duration (T_TOT_) of breaths with prolonged expiratory duration (T_E_), but not the characteristic shape of an augmented breath; during the baseline (BL) and fentanyl (F) periods. *n* = 32 animals (all animals in study pooled together). All comparisons were made using a paired sample two-tailed *t*-test.

Each breath was assigned to one of the twenty-one clusters identified in Figure 1 (see section “Data Analysis and Waveform Processing” for details). Clustering the waveforms allowed for automated annotation of the airflow trace throughout the experiment. This is indicated by the classification of waveforms as “sniffing,” “tidal breaths,” etc., in Figure 3A. The prevalence of each cluster was then evaluated within sequential windows consisting of 20 consecutive waveforms. The time period for each window was annotated if the majority (>51%) of waveforms met one of the inclusion criteria defined subsequently. To determine which tidal breaths were common during quiet or “eupneic” breathing, we calculated the prevalence of the tidal clusters (clusters 5–15) (i.e., durations of 0.2–1.2 s), during the baseline recording period across all animals. The clusters that occurred above average are shown in Supplementary Figure 2. Clusters 4, 6, 11, 12, and 14 were most commonly observed and were therefore marked as “common tidal breaths.” Any 20-breath window with a majority of breaths occurring from clusters 7, 8, 9, 10, 13, or 15 were marked as “uncommon tidal breaths.” If the majority of breaths within any 20-breath window did not fit these criteria (i.e., contained a mix of different behaviors), that time period was not annotated.

Figure 3 illustrates how assigning the breath waveforms to clusters allows flow traces to be annotated based on commonality in breath patterning, rather than simple metrics like V_T_ and rate. To evaluate the impact of fentanyl, tidal breaths were assessed in two categories; those that were common during the baseline period, and those that were uncommon at baseline. The incidence of the common tidal breaths was considerably lower during the fentanyl period (*p* < 0.0001, Figure 3B) and the prevalence of the uncommon tidal breaths increased during the fentanyl period (*p* < 0.0001, Figure 3C). The prevalence of short duration waveforms, presumably representative of sniffing activity (clusters 1–3) was also reduced following fentanyl injection (Figure 3D).

Augmented breaths (belonging to cluster 19) are annotated with an asterisk in the example traces shown in Figure 3A. The shape (Figure 3E) and prevalence (Figure 3F) of these augmented breaths were compared during the baseline and fentanyl periods. Fentanyl injection had no impact on the temporal appearance of the augmented breath waveform, however, the prevalence of augmented breaths was considerably reduced (*p* < 0.0002 Figure 3F). The other waveform clusters within the 1.2–4 s time domain all contained prolonged periods of no airflow (i.e., spontaneous apneas). The prevalence of these waveforms was increased after fentanyl injection (*p* < 0.0002), but the average breath length (T_TOT_) did not change (*p* > 0.14, Figure 3G).

### Impact of Ampakine CX1942 on Breathing After Opioid Overdose

**FIGURE 4:**
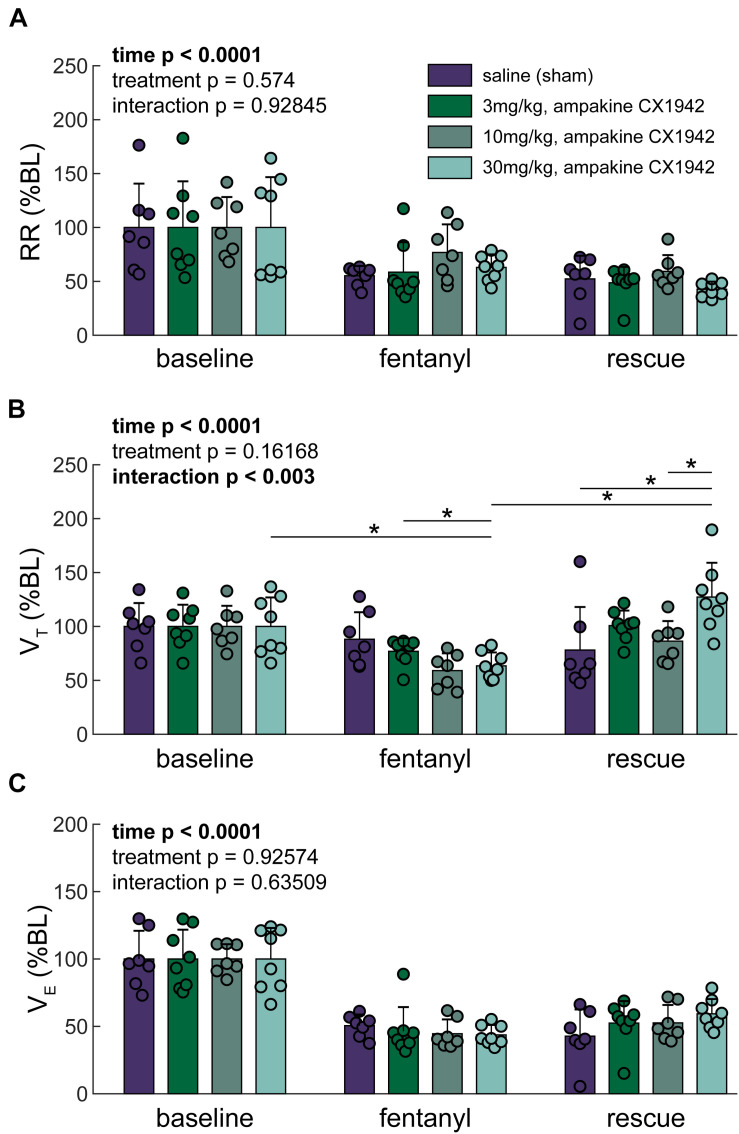
Traditional ventilation metrics. **(A)** Respiratory rate (RR), **(B)** Tidal volume (V_T_), and **(C)** Minute ventilation (V_E_) expressed as a percentage of baseline. Two-way ANOVA. *Post hoc* comparisons (Tukey–Kramer) are shown (*p < 0.05) if the interaction *p* < 0.05; further only the comparisons within a time point across groups, or within a group but across time points are shown. *n* = 8 animals/treatment group; 32 animals stratified by treatment condition.

Breathing was initially assessed using the traditional analyses of inspiratory V_T_ and respiratory rate (Figure 4). This approach confirmed that there was no difference between the four experimental groups at baseline (Supplementary Table 1). Fentanyl infusion caused the expected reduction of the respiratory rate (Ren et al., 2009) and none of the CX1942 doses corrected this (Figure 4A). Fentanyl also caused a reduction in V_T_. Figure 4B shows that all three of the CX1942 doses produced a tendency for greater V_T_ after opioid overdose, and in the high dose group (30 mg/kg), V_T_ values were restored to the pre-fentanyl baseline. The overall minute ventilation was depressed by fentanyl, but was not restored to baseline values by any of the three CX1942 doses (Figure 4C).

**FIGURE 5:**
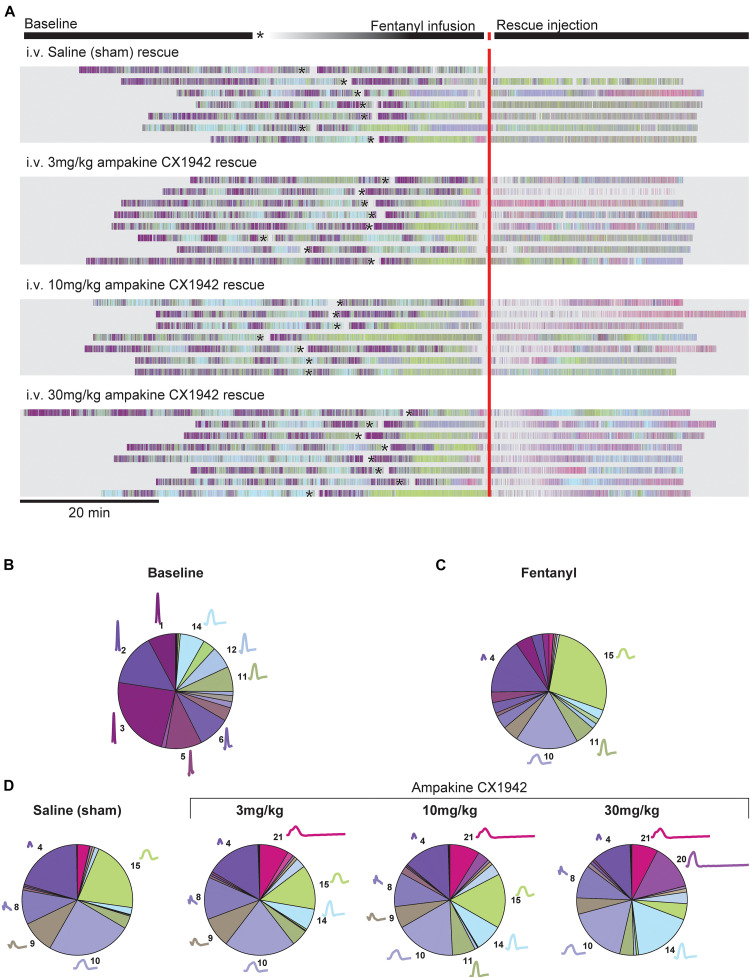
Cluster prevalence throughout experimental protocol stratified by rescue condition. **(A)** Peri-event raster plot, aligned to the rescue injection. Each line in the raster plot represents a single animal in each experimental condition. Rasters mark the beginning of inspiration for each breath; raster colors match cluster colors from Figure 1. The beginning of the fentanyl infusion is marked with an asterisk (*). Two animals excluded from plot due to prolonged fentanyl infusion period. The proportion of each cluster during the **(B)** baseline (*n* = 32, all animals in study pooled together), **(C)** fentanyl (*n* = 32, all animals in study pooled together), **(D)** rescue periods (*n* = 8 animals/treatment group). The average waveform and cluster number is plotted next to the pie segment if the cluster prevalence was greater than 5%.

We next examined the breathing patterns in greater detail using the cluster methods outlined in Figure 1. Raster plots were created to illustrate the breath by breath patterning across the entire experimental paradigm for all animals as shown in Figure 5A. The coloring of the raster plots corresponds to the waveform colors that are shown Figure 1F. Prior to fentanyl injection, during the baseline recording period, considerable variability in the breathing patterns can be observed. Thus, all rats showed baseline breathing waveforms corresponding to multiple clusters, and frequently transitioned between breaths belonging to different clusters. After infusion of fentanyl, however, there was a rapid transition, in all rats, to breaths which fell primarily into cluster 15 (Figures 2C, 5A, green). Reliance on this pattern continued throughout the entire recording session in seven of nine control animals that received the saline (i.e., sham) injection after fentanyl-induced hypoventilation. In contrast, when rats were treated with ampakine CX1942 the breathing pattern was no longer dominated by cluster 15. This can be appreciated by the abrupt shift from green raster lines to pink, purple, and light blue as shown in Figure 5A. This happened across all three doses tested.

Figures 5B–D provides a summary of how the waveform clusters changed after fentanyl and then ampakine CX1942 treatment. Fentanyl infusion very clearly reduces the variance observed during baseline, with only four clusters occurring more than 5% of the time. Clusters 4, 10, and 15, in particular, dominate the breathing after fentanyl (Figure 5C). Saline administration had little impact on the prevalence of these clusters as shown in Figure 5D (left panel). Following ampakine CX1942 treatment, the occurrence of clusters 4 and 10 are reduced in a dose-dependent manner. In addition, the occurrence of clusters 14 and 20 are increased. Thus, ampakine CX1942 dramatically altered breathing after opioid overdose, but not always in a manner that could be detected with the analyses shown in Figure 4.

**FIGURE 6:**
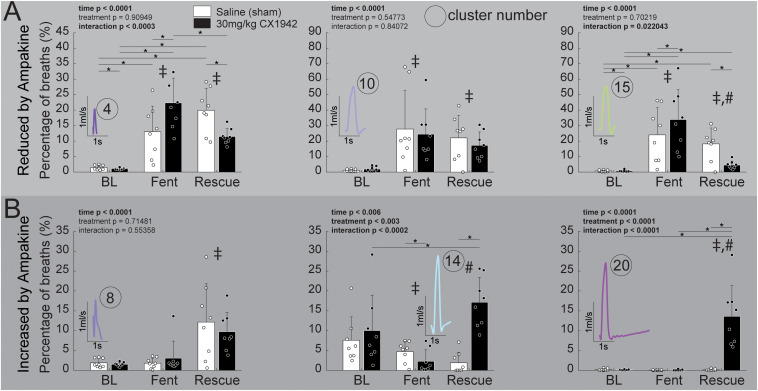
Prevalence of respiratory waveform clusters that were strongly affected by ampakine CX1942 injection. Comparison of the prevalence of clusters that were either **(A)** reduced (*n* = 8/treatment group) or **(B)** increased (*n* = 8/treatment group) by ampakine CX1942; between the control (saline) and high dose (30 mg/kg) ampakine groups. These six clusters were identified within the high dose (30 mg/kg) ampakine group as being affected by ampakine (see text). All comparisons were 2-way ANOVAs with Tukey–Kramer *post hoc* tests to control for multiple comparisons (*p < 0.05) were run if the interaction *p* < 0.05; if there was a significant (*p* < 0.05) main effect of time (BL, Fent, Rescue), a ‡ or a # indicates a significant (*p* < 0.05) pairwise comparison between baseline (BL) or fentanyl (Fent), respectively.

Since the high CX1942 dose (30 mg/kg) caused an increase in V_T_, we next assessed which clusters were most impacted by CX1942 in this group. Within the 30 mg/kg dose, six clusters were identified which had a >5% change in prevalence between the fentanyl and the CX1942 rescue periods. There were three clusters with reduced prevalence (clusters 4, 10, and 15), and three clusters (clusters 8, 14, and 20) with increased prevalence. Figure 6A demonstrates that clusters 4, 10, and 15 were nearly absent during baseline in both the sham and high dose CX1942 groups (<5% of breaths). Fentanyl infusion increased the prevalence of these clusters in both groups, indicating that breaths with these waveforms are phenotypical of opioid-induced respiratory depression. During the CX1942 rescue period, the animals in the control group maintained a prevalence of 18.2 ± 10.3% for cluster 15, while the animals in the ampakine CX1942 group only had a prevalence of 4.5 ± 2.5% (Figure 6B). For cluster 14, the prevalence during baseline was 7.6 ± 5.9% and 10 ± 8.9% for control and high dose groups, respectively, and these values were reduced to 4.6 ± 2.6% and 2.3 ± 2.9% during fentanyl injection. During the CX1942 rescue period, the prevalence of cluster 14 was further reduced in the saline treated group, but increased to 17.1 ± 6.3% in the high dose CX1942 group. Ampakine CX1942 injection also led the presence of a waveform cluster (cluster 20), which was rarely present at earlier time points (Figure 5D). Overall, ampakine CX1942 appears to restore aspects of normal ventilation (e.g., increased presence of cluster 14 and reduction of cluster 15). One interesting result in regards to ampakine CX1942 dose is that the low and mid-doses reduced the prevalence of cluster 15, whereas the high dose brought the prevalence of cluster 15 toward baseline.

The prevalence of six clusters identified to be impacted by high-dose ampakine CX1942 did not differ between the low (3 mg/kg) and mid-dose (10 mg/kg) groups at any time point (Supplementary Figure 3). However, both doses of ampakine CX1942 reduced the prevalence of cluster 15, 22–24% during fentanyl to 11–14% during rescue. There was an effect of time in five of the six clusters, with fentanyl increasing the cluster prevalence for clusters 4, 10, and 15 which were the clusters whose prevalence was reduced by high-dose ampakine CX1942 (Supplementary Figure 3). There was only a modest effect within the three clusters reduce by high-dose ampakine CX1942 with both the low and mid-dose increasing the prevalence of cluster 20 which was nearly absent in the time periods without ampakine CX1942 (Supplementary Figure 3).

## Discussion

Whole body plethysmography is a widely used method for monitoring respiratory rate and depth in preclinical studies of neuromuscular disease and/or respiratory motor control. Here we report waveform cluster analysis method that embraces the variability of WBP data and provides for evaluation of temporal changes in respiratory waveforms. The method effectively sorted WBP waveforms into categories including sniffing, tidal breaths of varying duration, and augmented breaths. By evaluating the entirety of a data record, versus picking an arbitrary period, the method enables assessment of how breathing is happening throughout an experimental paradigm. The waveform cluster analysis method also detected variations in the appearance of respiratory waveforms after opioid overdose and therapeutic intervention that were not evident from analyses of rate and/or amplitude measures.

### The WBP Method and Associated Respiratory Waveforms

The WBP method gained traction in respiratory research after the seminal report from Drorbaugh and Fenn (1955), and has been comprehensively reviewed previously (Enhorning et al., 1998; Mortola and Frappell, 1998). The waveforms that are recorded during WBP have limited value in regards to understanding lung mechanics, but can provide insight into the control of breathing and can provide reasonable estimates of respiratory volumes (Bates and Irvin, 2003). The pressure signal recorded during WBP results from a combination of: (1) gas compression and rarefaction, and (2) heating and humidification (Lundblad et al., 2002). The WBP signal is also impacted by upper and lower airway resistance as well as the rate of pressure changes. The aforementioned variables contribute to the variability of the recorded WBP signal. Further, factors such as body weight and temperature (Mortola and Frappell, 1998) can affect the recorded waveforms, and these variables need to be considered when comparing the waveform patterns. The central thesis of the current work is that the diversity of respiratory waveforms recorded during WBP creates a need, and an opportunity, for comprehensive evaluation of the all of the recorded signals.

The difference between body temperature and chamber temperature may be a confounding factor in the present study. Prior work establishes that the amplitude of the WBP signal decreases as body temperature approaches chamber temperature (Malan, 1973; Mortola and Frappell, 1998). In our experiments we observed a slight drop in body temperature (−0.4°C) over the entire recording period that was similar in all animals. The effect that these small changes in body temperature will have on the shape of waveforms is unknown, and could impact the interpretation of the effects of fentanyl and ampakines. However, the temperature drop was similar in all of the experimental groups, and we suggest it is unlikely playing a significant role in the between group comparisons during the ampakine rescue period.

### Analyses Approaches to Respiratory Waveforms

The physiology literature has a rich history of respiratory waveform evaluation. Review of early work shows kymograph recordings of thoracic pressures and/or movements used to measure the rate and estimate the depth of breathing (Porter, 1895). Caughey (1943) expanded this slightly, concluding that analysis of breathing patterns, and not exclusively rate and volume, could help understand pathophysiology. However, most evaluations of respiratory waveforms, even over recent decades, have continued to focus on amplitude/volume and rate since these parameters provide a fundamental description of how breathing is occurring. Exceptions include WBP waveforms that have been processed to detect apneic events (Matrot et al., 2005) or breath to breath variability (Ermer et al., 2020). For example, breathing waveforms in humans with opioid overdose were analyzed with Poincaré plots, and a machine learning algorithm (Ermer et al., 2020). The authors concluded that the algorithm could identify “ataxic” breathing patterns as well as human experts. Tobin et al. (1988) were among the first to evaluate variability of respiratory waveforms in humans. They evaluated the coefficient of variation, and concluded that tidal volume shows greater breath-to-breath variability than rate in the spontaneously breathing human (Tobin et al., 1988). Work summarized in a review by Benchetrit (2000) suggests that humans may have unique breathing patterns that persist over time and assessment of these patterns could be used to identify individuals (Proctor and Hardy, 1949). This interesting possibility was not directly addressed in our experiments. However, in our data there were different patterns over time and between animals. This is evidenced in the raster plots, which show how the animals produced respiratory waveforms associated with different clusters throughout the experimental protocol. Specifically, during baseline breathing most animals would switch between periods that consisted largely of waveforms associated with clusters 1–3, 5, and 6, and then periods dominated by clusters 11, 12, and 14. However, the exact pattern (e.g., duration utilizing a set of cluster, or clusters utilized during transitions) varied between animals. These breath to breath or cluster to cluster variations produced patterns over time utilizing waveforms from the same set of 21 clusters. Further, signal processing techniques and cluster analyses have also been used identify respiratory impairments that are not captured by reduction in V_T_ (Garde et al., 2007, 2011, 2017; Aydore et al., 2009). For example, Garde et al. (2017) used characteristics of the respiratory flow cycle to detect periodic breathing in congestive heart failure (CHF) patients. A sliding 30 s window was used to create a template of a representative respiratory cycle. Parameters such as inspiratory and expiratory duration and waveform slopes were then determined from the template. This work demonstrated that analysis of the respiratory flow cycle “morphology” could provide clinically relevant information about CHF patients (Garde et al., 2017). The current study builds upon the foundation of prior work with evaluation of respiratory waveforms, in particular aforementioned work by Garde et al. (2011). Perhaps the most novel aspect of the current work is that we used the entirety of the recorded data set to build respiratory waveform templates. When applied across multiple experiments, this produced a sample of more than 300,000 respiratory waveforms for evaluation using principle component analyses. Further, we were able to visualize and categorize an entire data set (e.g., raster plots in Figure 5). This enables automated annotation of breath types and comprehensive evaluation of breathing across time. This approach to evaluation of plethysmography data does not use pre-selected components of the respiratory waveform or selective identification of arbitrary “baseline” periods. Further, the method was able to identify waveforms that may be artifacts (e.g., clusters 4 and 17). Within the current data set, we had no method to definitively confirm if these waveforms were related to breathing, or were truly an artifact. However, the low amplitude and short duration indicates that they may be artifact. On the other hand, the prevalence of cluster 4 increased during the fentanyl administration when the animals were less mobile and semi-sedated. While we could not confirm the source of these particular waveforms, the salient point is that the analysis method is sensitive enough to stratify the data and could allow for removing of artifacts from data records to enable more accurate calculations of tidal volume, rate, and minute ventilation. Importantly, our method of assessing respiratory waveforms can be adapted to produce a specific number of clusters depending on the question of interest (i.e., the number of clusters can be expanded such that a particular behavior known to exist within a disease model is evident in the cluster waveform averages). In fact there is not a consensus on how to identify the number of clusters within a dataset, the number of clusters will vary based on field of study and research question (Thorndike, 1953).

One potential limitation of applying the waveform cluster analysis method across data sets is the sensitivity to the amplitude of the respiratory waveforms. Specifically, the amplitude can drive assignment of waveforms to different clusters. While this is appropriate within a given data set, it could cause problems when comparing different data sets. However, if WBP recording chambers are appropriately calibrated, clustering should be accurate across datasets from different systems or laboratories. Further, laboratories can maintain a training set of waveforms from prior experiments, this training set can be used to fit new data within a framework of established clusters in order to compare newly collected data to older experiments.

### Opioids and Ampakine Rescue

Opioids act on mu-opioid receptors throughout the brainstem in areas that control respiration (Mansour et al., 1994; Pattinson, 2008). It is well established that activation of mu-opioid receptors can lead to severe respiratory depression (Downes et al., 1967; Dahan et al., 2010), and this is the leading cause of death in the ongoing opioid public health crisis (Dahan et al., 2010; Bedene et al., 2020; Bird and Robertson, 2020). Additionally, mu-opioid receptor mRNA is present throughout the spinal cord, primarily in the dorsal horns (Mansour et al., 1994; Peckys and Landwehrmeyer, 1999; Schnell and Wessendorf, 2008), and has been observed in the spinal ventral horn (Peckys and Landwehrmeyer, 1999) and spinal motor neurons (Mansour et al., 1994). These could provide additional sites of action to suppress respiration, but it is unknown if spinal respiratory neurons express mu-opioid receptors.

Here we used the new waveform cluster analysis method to explore the impact of ampakine CX1942 on breathing after opioid induced respiratory depression. Ampakines are class of drugs that alter AMPA receptor channel kinetics (Lynch, 2006; Arai and Kessler, 2007) and can act as respiratory stimulants as well as enhancing expression of respiratory neuroplasticity (Wollman et al., 2020). The low impact ampakine CX717 can attenuate opioid induced respiratory depression in rodents (Ren et al., 2009) as well as humans (Oertel et al., 2010). However, CX717 has relatively limited solubility and requires a solvent such as 2-hydroxypropyl-β-cyclodextrin (HPCD) to go into solution. In contrast, ampakine CX1942 is soluble in water, which makes it a more attractive candidate for potential clinical use. Prior work showed that CX1942 (20 mg/kg) can effectively prevent hypoxemia in opioid (etorphine, 0.1 mg/kg) overdosed goats (Haw et al., 2016). Evaluation of the data in that study shows that CX1942 treatment was associated with a relatively large increase in V_T_ [baseline value: 0.28 ± 0.07 (SD) L, 15 min post-treatment: 0.57 ± 0.16 L] with relatively little impact on respiratory rate (Haw et al., 2016). Our data are consistent with the finding, since CX1942 at the highest dose (30 mg/kg) resulted in an increase in the volume of inspiratory efforts (Figure 4 and Supplementary Table 1). This finding raises interesting questions about the mechanism of action of CX1942. Prior researchers using ampakine CX717 suggested that its primary mechanism of action, in regards to reversing opioid overdose, was on respiratory rhythm generating neurons/networks (Ren et al., 2009, 2013; Oertel et al., 2010). The lack of impact on respiratory rate coupled with increased V_T_, however, suggests a different mechanism of action. One possibility is an impact on phrenic motor neurons, which robustly express AMPA receptors (Rana et al., 2019b).

Lastly, we observed that the new waveform cluster analysis method enabled detection of changes in the WBP data that were not seen with the traditional analyses approach. Fentanyl infusion caused rats to adopt breathing using primary three waveform types: a short, low amplitude effort (cluster 4), and two variants of low amplitude breathing of “normal” duration (clusters 10 and 14). Thus, we conclude that these breathing patterns are phenotypical of opioid-induced respiratory depression in the rat. Whereas saline administration (i.e., sham treatment) had no impact on the prevalence of these three clusters, treatment with CX1942 reduced the occurrence of clusters 4 and 10 in a dose-dependent manner. Further, CX1942 caused an increase in two specific clusters in the 0.5–1.2 s duration range. Interpreting the underlying physiology will require more in depth assessment such as respiratory muscle EMG and measures of airway resistance, etc. Nevertheless, these observations provide a framework for developing hypothesis driven questions about how overdose and or ampakine treatment alter the manner in which breathing is occurring.

## Conclusion

The principle component based method of respiratory waveform cluster analysis provides rapid assessment of respiratory signals and tracks common breath types across animals and experimental conditions. The analysis is able to detect changes in respiratory flow patterns associated with opioid (fentanyl) induced respiratory depression and ampakine CX1942 rescue. Since most, if not all, current researchers have access to sufficient computing power to conduct higher level analyses of WBP signals, and this approach using a widely available coding platform (MATLAB), could find widespread applicability. In summary, the method enables rapid assessment of breathing patterns; we suggest that this approach may prove useful in future studies of how breathing is affected in progressive neuromuscular diseases and/or during therapeutic interventions.

## Data Availability Statement

The raw data supporting the conclusions of this article will be made available by the authors, without undue reservation.

## Ethics Statement

The animal study was reviewed and approved by the University of Florida Institutional Animal Care and Use Committee.

## Author Contributions

MS conceived and coded the analyses methods, analyzed the data, and drafted the manuscript. DF designed the ampakine rescue experiments, interpreted the data, and provided feedback on the manuscript. Both authors contributed to the article and approved the submitted version.

## Conflict of Interest

The authors declare that the research was conducted in the absence of any commercial or financial relationships that could be construed as a potential conflict of interest.

## Publisher’s Note

All claims expressed in this article are solely those of the authors and do not necessarily represent those of their affiliated organizations, or those of the publisher, the editors and the reviewers. Any product that may be evaluated in this article, or claim that may be made by its manufacturer, is not guaranteed or endorsed by the publisher.
