# Supplementary material for: Automated Classification of Whole Body Plethysmography Waveforms to Quantify Breathing Patterns
Source: Front Physiol. 2021 Aug 20;12:690265. doi: 10.3389/fphys.2021.690265 (PMC8417563; doi:10.3389/fphys.2021.690265)
Supplement: Supplementary file 1 [file Data_Sheet_1.PDF]

Supplementary Material

SUPPLEMENTAL FIGURES AND TABLE

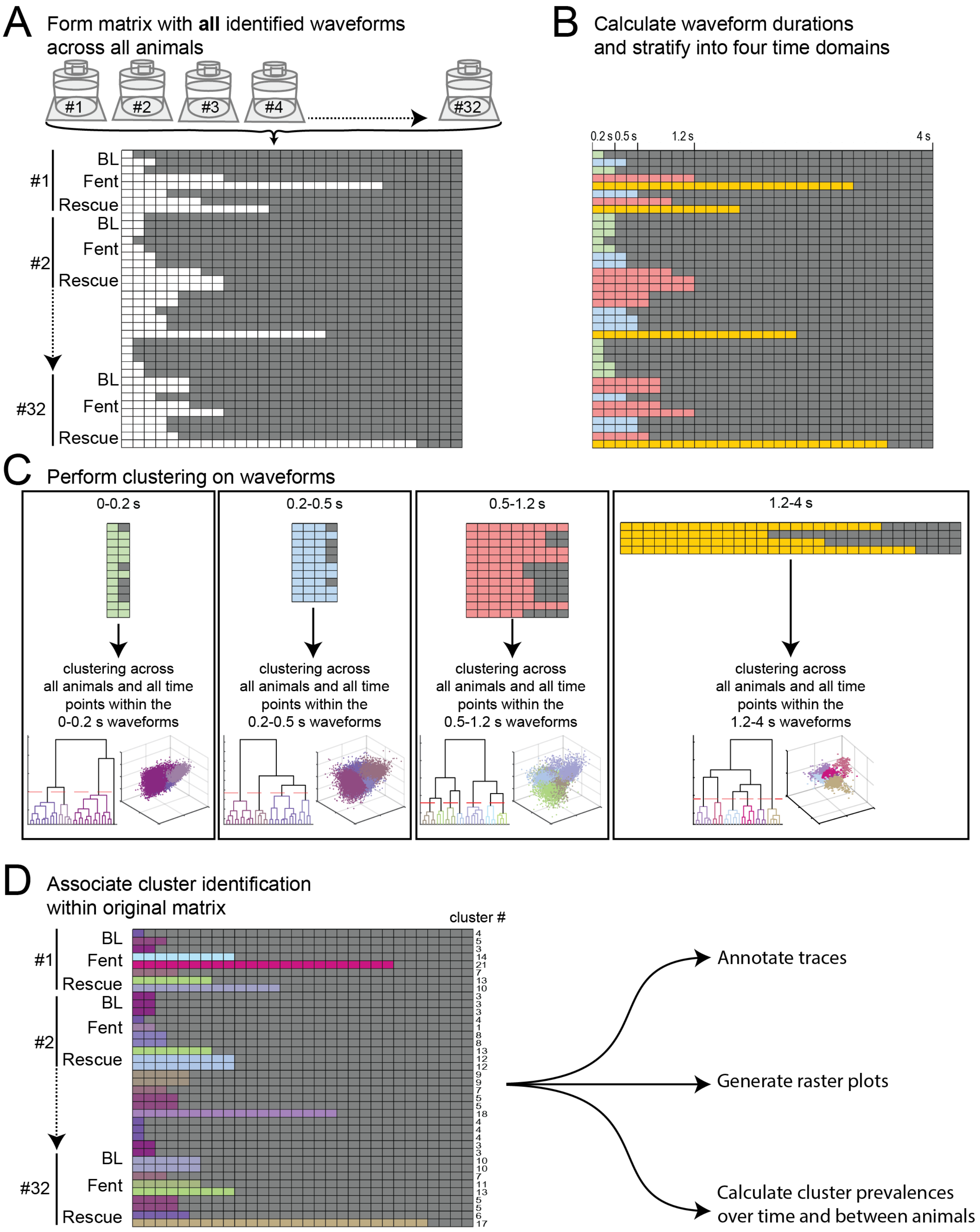

Supplemental Figure 1. Schematic diagram of the waveforms clustering process. A) Once a waveform was identified via threshold crossing, the waveform was inserted into a matrix that included every identified respiratory waveform. The schematic shown in panel A is an abbreviated table, the true table consisted of over 300,000 identified waveforms, from all animals and across the entire recording period including waveforms from baseline, fentanyl overdose, and rescue. In this schematic, each box represents 0.1 s (~12 samples) of data. The white boxes indicate the waveform length. In addition to the waveforms, vectors of equivalent length were generated that contained experimental identifiers such as animal identification, group assignment, and sample number at the beginning of inspiration. These identifiers were not used in the clustering process but were used later to track when particular clusters occurred. **B)** The length of each waveform was calculated to enable stratification into four time domains: 0-0.2 (green), 0.2-0.5 (blue), 0.5-1.2 (pink), or 1.2-4 seconds (yellow) **C)** We next performed cluster analysis once per time domain. A matrix consisting of waveforms from all animals within each time domain was run through principle component analysis to determine how many unique clusters existed within that matrix. Within the shortest time domain (0-0.2 s) the original matrix consisted of 127,602 waveforms, the analysis identified four clusters within this time domain. Within the 0.2-0.5, 0.5-1.2, and 1.2-4 second time domains the original matrices consisted of 67,584, 125,232, and 12,727 waveforms (including breaths from all animals), the analyses identified four, seven, and six clusters within the respective time domains. **D)** This analysis produced a cluster identification number for each waveform. The schematic matrix is color coded to illustrate that waveforms are now identified as belonging to a particular cluster. An array of cluster identifiers was also generated. Each value in this array corresponded to a waveform and we then referenced the arrays of animal identification, group assignment, and sample to quantify how frequently waveforms belonging to particular cluster occurred (e.g. frequency between baseline and fentanyl overdose, or between the saline and 30 mg/kg ampakine group).

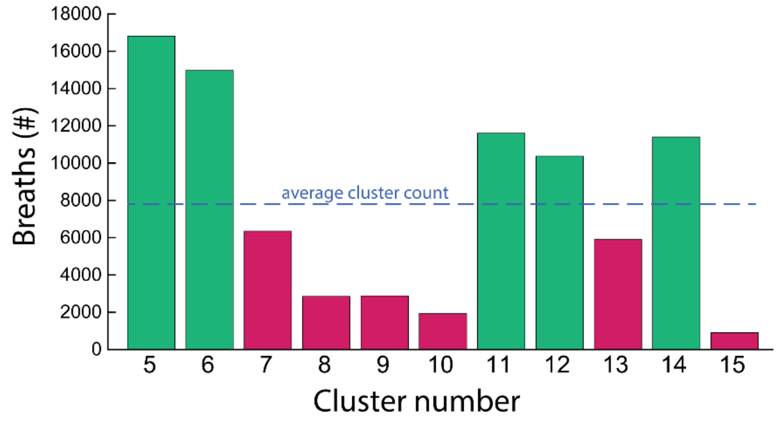

Supplemental Figure 2. Cluster prevalence under baseline conditions. Clusters 5-15 were classified as tidal breaths because their durations ranged from 0.2-1.2 seconds. The average prevalence for tidal clusters (dashed blue line) was 7796 breaths. Clusters 5, 6, 11, 12, 14 occurred more often and were classified as "common tidal breaths at baseline", while clusters 7, 8, 9, 10, 13, and 15 occurred less often and were classified as uncommon.

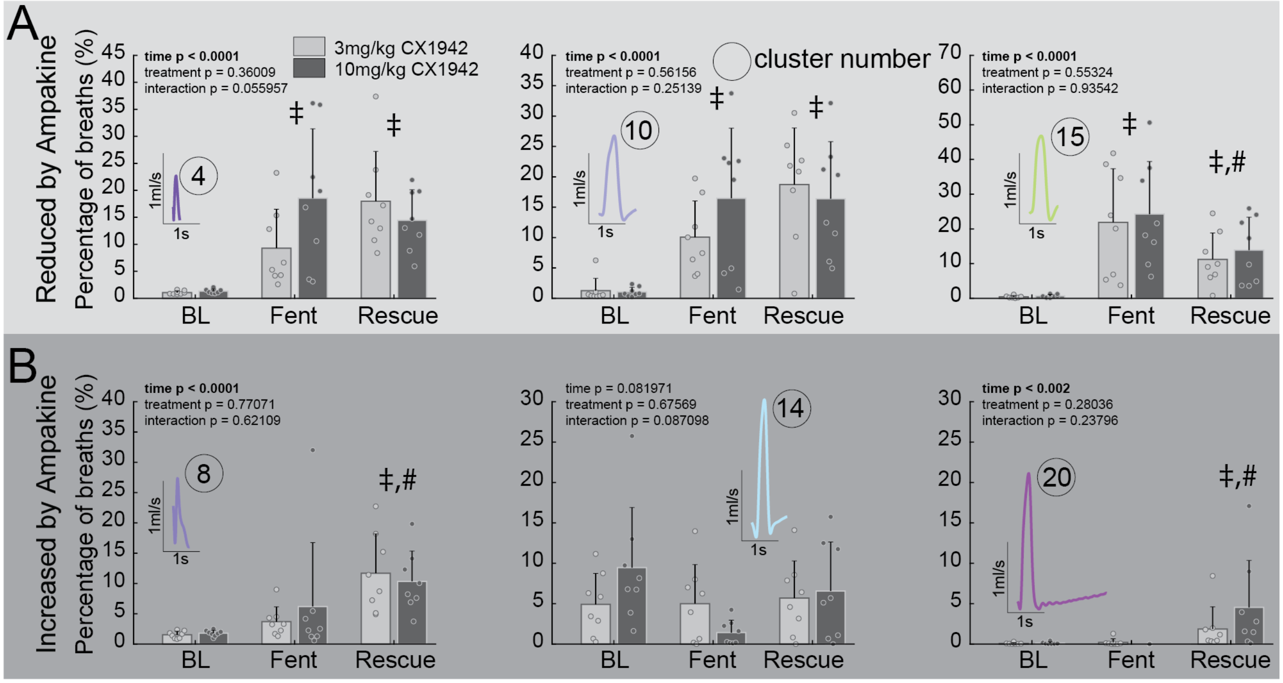

Supplemental Figure 3. Prevalence of respiratory waveform clusters that were strongly affected by ampakine CX1942 injection. Comparison of the prevalence of clusters **A)** reduced (n=8/ treatment group) or **B)** increased (n=8/ treatment group) by ampakine between the low dose (3 mg/kg) and mid-dose (10 mg/kg)

ampakine groups. These six clusters were identified within the high dose (30 mg/kg) ampakine group as being affected by ampakine (see text). All comparisons were 2-way ANOVAs Tukey-Kramer post-hocs to control for multiple comparisons were run if the interaction $p < 0.05$; if there was a significant ($p<0.05$) main effect of time (BL, Fent, Rescue), a ‡ or a # indicates a significant ($p<0.05$) pairwise comparison between baseline (BL) or fentanyl (Fent) respectively.

	**Respiratory Rate (bpm) (mean ± SD)**			**Tidal Volume (ml) (mean ± SD)**			**Minute Ventilation (ml/min) (mean ± SD)**		
	**Baseline**	**Fentanyl**	**Rescue**	**Baseline**	**Fentanyl**	**Rescue**	**Baseline**	**Fentanyl**	**Rescue**
**Saline (sham)** rescue	144.9 ± 62.1	84.8 ± 40.3	70.6 ± 21.4	0.337 ± 0.068	0.259 ± 0.041	0.34 ± 0.047	45.3 ± 9.9	21 ± 8.1	23.8 ± 7.4
**3 mg/kg** CX1942 rescue	122.2 ± 34.5	93.9 ± 31.9	72.5 ± 18.	0.353 ± 0.068	0.209 ± 0.057	0.305 ± 0.066	41.3 ± 4.5	18.4 ± 4.4	21.7 ± 5.5
**10 mg/kg** CX1942 rescue	146.7 ± 59.7	81.1 ± 13.2	76.8 ± 31.4	0.333 ± 0.072	0.294 ± 0.084	0.26 ± 0.133	45.6 ± 9.5	23 ± 3.8	19.5 ± 9
**30 mg/kg** CX1942 rescue	149.7 ± 69.9	94.2 ± 18.2	64 ± 10.5	0.357 ± 0.096	0.227 ± 0.044	0.455 ± 0.113	47.9 ± 11	20.9 ± 3.6	28.4 ± 5.2

Supplemental Table 1. Traditional ventilation metrics. Respiratory rate, tidal volume, and minute ventilation across the experimental protocol.
